# Early Versus Late Onset of Cannabis Use: Differences in Striatal Response to Cannabis Cues

**DOI:** 10.1089/can.2016.0026

**Published:** 2016-10-01

**Authors:** Reagan R. Wetherill, Nathan Hager, Kanchana Jagannathan, Yasmin Mashhoon, Heather Pater, Anna Rose Childress, Teresa R. Franklin

**Affiliations:** ^1^ Department of Psychiatry, University of Pennsylvania, Philadelphia, Pennsylvania.; ^2^ McLean Hospital/Harvard Medical School, Belmont, Massachusetts.

**Keywords:** cannabis, cues, age of onset, early onset, craving

## Abstract

Addiction theories posit that addiction is the result of a progressive transition from voluntary to habitual, compulsive drug use—changes that have been linked, in animals, to a shift from ventral to dorsal striatal control over drug-seeking behavior. Thus, we hypothesized that early-onset (EOs) cannabis users versus late-onset (LOs) cannabis users might exhibit, respectively, greater dorsal versus ventral striatal response to drug cues. We used functional magnetic resonance imaging and an event-related blood oxygen level-dependent backward-masking task to evaluate striatal responses to backward-masked cannabis cues (vs. neutral cues) in EOs (<16 years old, *n*=15) and LOs (≥16 years old, *n*=26) with similar recent cannabis use patterns. Direct comparisons revealed that EOs showed greater response to cannabis cues in the dorsal striatum than LOs (*p*<0.01, *k*>50 voxels). Within-group analyses revealed that EOs showed greater neural response to cannabis cues in the dorsal striatum, whereas LOs exhibited greater neural response to cannabis cues in the ventral striatum. Although cross-sectional, these findings are consistent with recent addiction theories suggesting a progressive shift from ventral to dorsal striatal control over drug-seeking behavior and highlight the importance of age of onset of cannabis use on the brain and cognition.

## Introduction

Globally, cannabis is the most widely used psychoactive substance.^1^ In the United States, ∼22.2 million people aged 12 or older report using cannabis in the past month,^2^ and numbers will likely increase as states continue to legalize cannabis for medicinal and recreational purposes.^3^ Potential increases in cannabis use rates are concerning, as research suggests that cannabis use, particularly during adolescence and early adulthood, contributes to atypical brain structure and function, as well as cognitive deficits.^4–8^

Although cannabis use may lead to alterations in the brain and cognition, these changes occur over time. Indeed, addiction theories posit that substance use disorders are the result of “a series of transitions from initial voluntary drug use to habitual, and ultimately compulsive drug use”^9^ (p.1946). These transitions include a shift from ventral to dorsal striatal control over behaviors (and impaired prefrontal inhibitory control) that contribute to habitual and progressively compulsive drug seeking.^10–12^ The majority of the research supporting this theory has been conducted in animal models; however, recent human studies on age of onset of cannabis use may provide additional support for such brain changes and impairments in prefrontal inhibitory control. Specifically, early (before age 16) onset of cannabis use (EO) has been associated with structural connectivity differences of the orbitofrontal cortex (OFC),^13^ decreased white matter integrity in fibers connecting the right and left dorsolateral prefrontal cortex,^14^ and increased functional connectivity between the OFC and prefrontal and motor regions.^15^ Furthermore, research indicates that EO cannabis users (EOs) perform worse than late-onset (age 16 or later) cannabis users (LOs) across a variety of neurocognitive domains, including measures of sustained attention, impulse control, and executive functioning.^5,6,16,17^ Together, these findings suggest that EO of cannabis use may contribute to morphological alterations in prefrontal brain regions that are associated with cognitive control deficits observed in EOs.

While morphological and cognitive changes have been well characterized in EOs, it remains unknown whether age of cannabis use onset is differentially associated with ventral or dorsal striatal activity or control over cannabis use. Thus, the current study explored potential differences in striatal activity during cannabis cue exposure (compared to neutral cues) among EOs and LOs who report similar patterns of cannabis use. We compared striatal responses to cannabis cues presented during a functional magnetic resonance imaging (fMRI) backward-masked cannabis cue paradigm^18,19^ and investigated the associations between striatal activity and cannabis craving. Based on the research described above, we expected that EOs would show neural response to backward-masked cannabis cues (compared to neutral cues) in the dorsal striatum, whereas LOs would show neural response to cannabis cues in the ventral striatum. Because research suggests that heightened motivational/emotional states (e.g., craving) lead to heightened sensitivity to associated cues,^20^ we also hypothesized that cannabis craving would correlate with these cannabis cue-related activations and that different association patterns would emerge in EOs and LOs.

## Materials and Methods

### Participants

All study procedures adhered to the Declaration of Helsinki and were approved by the University of Pennsylvania Institutional Review Board. Details of the recruitment process and selection criteria were reported previously.^18,19^ Participants were 41 treatment-seeking individuals who met the DSM-IV^21^ criteria for cannabis dependence. Participants were medically stable, educated, and had no concomitant serious comorbid psychiatric or substance use disorders (except nicotine dependence). See Table 1 for participant demographics.

**Table 1.** **Participant Characteristics**

*Measure*	*Early onset (*n*=15)mean (SEM)*	*Late onset (*n*=26)mean (SEM)*	p
Age	29.0 (2.0)	28.7 (1.4)	0.91
Sex (% male)	40%	73%	χ^2^*p*=0.04
Ethnicity (% African American)	73%	77%	χ^2^*p*=0.41
Education (Years)	12.5 (0.4)	13.1 (0.3)	0.20
Age of onset of cannabis use	13.1 (0.6)	19.5 (0.8)	0.00
Gram years (lifetime use)	38.0 (23.5)	28.3 (34.0)	0.35
Cannabis craving	44.9 (3.7)	39.0 (3.0)	0.23
Substance use in the last 30 days			
Cannabis grams daily	3.3 (0.5)	3.5 (0.9)	0.85
# EtOH days	2.1 (0.9)	4.0 (0.9)	0.15
# EtOH drinks per day	5.7 (2.4)	4.1 (0.6)	0.53
# Cigarette days	17.0 (3.7)	12.3 (2.8)	0.32
# Cigarettes per day	2.5 (0.7)	2.9 (0.7)	0.71

SEM, standard error of mean.

As part of a larger study, participants completed baseline questionnaires, interviews, and an MRI session. Participants were asked to abstain from alcohol and illicit substances for the 24 h before the MRI session and completed a urine drug screen and alcohol breathalyzer. All participants were positive for cannabis use and negative for other substance use.

### Measures

Cannabis, alcohol, and other drug use during the preceding 30 days was assessed with the Timeline Follow-Back interview,^22^ and the Addiction Severity Index^23^ measured lifetime alcohol and substance use and age of onset of cannabis use. Based on the existing literature and that age 16 is when significant brain changes typically occur,^24^ participants were grouped as EO (<16 years old) or LO (≥16 years old). Lifetime cannabis exposure was quantified using gram years.^25^ The Marijuana Craving Questionnaire-Short Form (MCQ-SF)^26^ measured self-reported baseline cannabis craving using a scale covering behavioral experiences associated with aversive and appetitive aspects of drug motivation. The magnitude of cannabis craving was assessed before the neuroimaging session and was determined by summing items of the MCQ-SF.

### Analyses

A detailed description of the backward-masking cannabis cue paradigm, imaging parameters, and analyses are provided in previous publications.^18,19^ Briefly, imaging data were analyzed using statistical parametric mapping (SPM8; Wellcome Department of Cognitive Neurology, London, United Kingdom). Imaging analyses were focused on regions of interest (ROIs) in the dorsal and ventral striatum. The ROIs were created using the Harvard–Oxford probabilistic anatomical atlas provided with the FMRIB Software Library.^27^ To control for type 1 error, neural activity within the ROI mask of each voxel was considered significant at a nominal alpha level of *p*<0.01 and a cluster extent of 50 contiguous resampled voxels as determined via Monte Carlo simulations using *3dClustSim* Analysis of Functional NeuroImages software^28^ (http://afni.nimh.nih.gov/).

## Results

As noted in Table 1, EOs and LOs did not differ significantly on age, alcohol use, cigarettes per day, or cannabis use. Chi-square analyses revealed significant difference in the number of males and females in the EO and LO groups (χ^2^ [1, *N*=41]=4.37, *p*=0.04); however, small sample size prevented the exploration of potential sex differences in the current analyses.

**FIG. 1.**
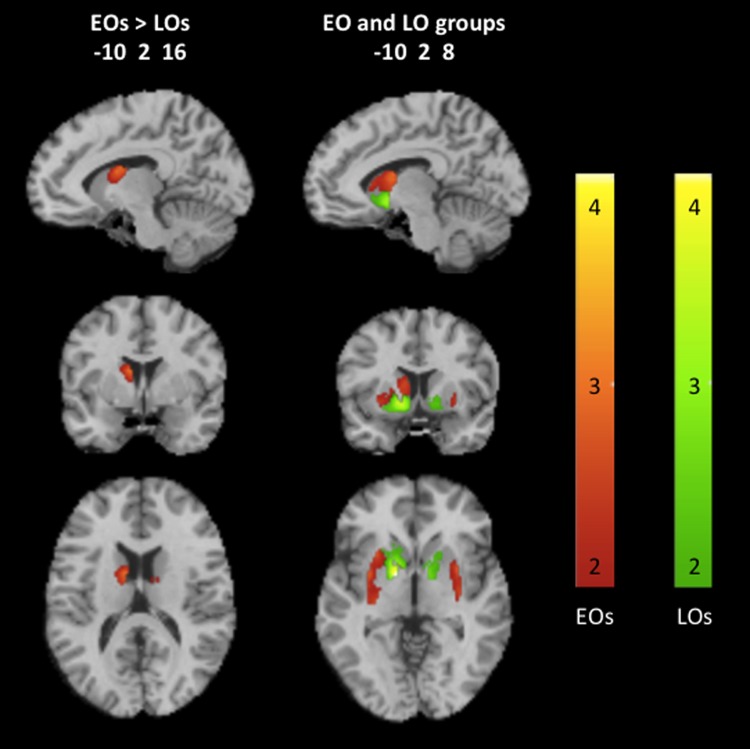
Neural responses to backward-masked cannabis cues compared to neutral cues in the striatum of early and LO cannabis users (*p*<0.01, *k*>50 voxels). Representative fMRI sagittal, axial, and coronal brain slices analyzed in SPM8 and overlain on the MNI brain. Data are displayed neurologically (left is left). The color bars, which represent T values, indicating greater dorsal striatal responses in EOs are shown in red to yellow hues, and greater ventral striatal responses in LOs are shown in green to yellow hues. An interactive visual display of all brain data in all three planes can be found at http://franklinbrainimaging.com. EO, early onset; LO, late onset; SPM8, statistical parametric mapping.

Direct comparisons of neural responses to backward-masked cannabis cues compared to neutral cues between EOs and LOs revealed that EOs showed significantly greater response in the dorsal striatum than LOs (*p*<0.01, *k*>50 voxels). Analyses among EOs revealed greater response to backward-masked cannabis cues in the dorsal striatum, whereas LOs showed greater response to backward-masked cannabis cues in the ventral striatum (Fig. 1).

Correlation analyses between cannabis craving scores and *β* coefficients extracted from the functional clusters from the cannabis cue versus neutral cue contrast in the ventral and dorsal striatum revealed a positive correlation in EOs between cannabis craving and the dorsal striatal clusters showing activation to backward-masked cannabis cues (*r*[15]=0.52, 0.56, 0.66, *p*s<0.05). There were no significant correlations among LOs.

## Discussion

As hypothesized, preliminary analyses revealed that EOs and LOs showed different patterns of neural response to backward-masked cannabis cues, with EOs showing greater response within the dorsal striatum compared to LOs. When examining groups separately, EOs exhibited neural response to backward-masked cannabis cues in the dorsal striatum, yet LOs exhibited neural response in the ventral striatum. It is important to note that the EO and LO groups reported similar patterns of recent cannabis use, and as such, findings are not due to differences in recent cannabis use. Furthermore, groups did not show significant differences in lifetime cannabis use, suggesting that striatal activation findings in EOs and LOs were specific to age of onset of cannabis use and not due to history of daily use or duration of use. Correlation analyses revealed that dorsal striatal activations in EOs correlated with cannabis craving. Although cross-sectional, these preliminary findings are the first of their kind and suggest that differential striatal activation between EOs and LOs may reflect cannabis-induced alterations in neuroplasticity during early adolescent maturation that strengthened reward-related associations in EOs and possibly accelerated the shift from voluntary occasional use to habitual compulsive use.

Our findings, in conjunction with previous research demonstrating altered prefrontal structure and function,^4–8,13–15^ provide evidence that EOs also exhibit striatal brain changes that may underlie habitual, compulsive cannabis use, whereas LOs exhibit a pattern of neural activity in the ventral striatum that is characteristic of voluntary cannabis use, which may be regulated by prefrontal control processes. Although compulsivity and impulsivity were not assessed in the current study, future research in a larger sample could explore these hypotheses more fully.

Limitations should be considered when interpreting the findings. First, sample size is moderate; thus, future studies should include a larger sample with greater diversity to validate these findings and ensure generalizability. Furthermore, we did not assess or control for the influence of menstrual cycle phase/gonadal hormones, cannabis withdrawal symptoms, or motivations for treatment, and as such, it remains unclear as to whether these factors influenced findings. Finally, this study used a cross-sectional design, so it is not possible to know whether EOs exhibited ventral striatal activation earlier in their addiction. Longitudinal studies will be helpful in parsing the effects of these factors on cannabis cue reactivity.

In summary, EOs and LOs exhibited differential patterns of striatal response to backward-masked cannabis cues, with EOs demonstrating dorsal striatal response and LOs showing a ventral striatal response. This differential pattern of striatal response parallels recent hypotheses of drug addiction, through which a series of transitions from initial voluntary use to habitual, compulsive use involve transitions from ventral to dorsal striatal control. Although additional research is warranted, our findings are the first neuroimaging findings among cannabis users to demonstrate differential striatal responding to cannabis cues in EOs and LOs.

## Abbreviations Used

EOs: early-onset cannabis users
LOs: late-onset cannabis users
MCQ-SF: marijuana craving questionnaire-short form
OFC: orbitofrontal cortex
ROIs: regions of interest
SEM: standard error of mean
SPM8: statistical parametric mapping
